# Mitochondrial genome sequencing reveals potential origins of the scabies mite *Sarcoptes scabiei* infesting two iconic Australian marsupials

**DOI:** 10.1186/s12862-017-1086-9

**Published:** 2017-11-28

**Authors:** Tamieka A. Fraser, Renfu Shao, Nicholas M. Fountain-Jones, Michael Charleston, Alynn Martin, Pam Whiteley, Roz Holme, Scott Carver, Adam Polkinghorne

**Affiliations:** 1 School of Biological Sciences, University of Tasmania, Sandy Bay, Hobart, TAS Australia; 2 Centre for Animal Health Innovation, School of Science and Engineering, University of the Sunshine Coast, Sippy Downs, QLD Australia; 3 University of Minnesota, Minneapolis, MN USA; 4 School of Information Technologies, University of Sydney, Camperdown, NSW Australia; 5 Faculty of Veterinary and Agricultural Sciences, The University of Melbourne, Werribee, VIC Australia; 6 Cedar Creek Wombat Rescue Inc. & Hospital, Cedar Creek, NSW Australia

**Keywords:** *Sarcoptes scabiei*, Wombat, Koala, Mitochondrial genome sequencing, *cox*1, Phylogeny, Conservation

## Abstract

**Background:**

Debilitating skin infestations caused by the mite, *Sarcoptes scabiei,* have a profound impact on human and animal health globally. In Australia, this impact is evident across different segments of Australian society, with a growing recognition that it can contribute to rapid declines of native Australian marsupials. Cross-host transmission has been suggested to play a significant role in the epidemiology and origin of mite infestations in different species but a chronic lack of genetic resources has made further inferences difficult. To investigate the origins and molecular epidemiology of *S. scabiei* in Australian wildlife, we sequenced the mitochondrial genomes of *S. scabiei* from diseased wombats (*Vombatus ursinus*) and koalas (*Phascolarctos cinereus*) spanning New South Wales, Victoria and Tasmania, and compared them with the recently sequenced mitochondrial genome sequences of *S. scabiei* from humans.

**Results:**

We found unique *S. scabiei* haplotypes among individual wombat and koala hosts with high sequence similarity (99.1% - 100%). Phylogenetic analysis of near full-length mitochondrial genomes revealed three clades of *S. scabiei* (one human and two marsupial), with no apparent geographic or host species pattern, suggestive of multiple introductions. The availability of additional mitochondrial gene sequences also enabled a re-evaluation of a range of putative molecular markers of *S. scabiei*, revealing that *cox1* is the most informative gene for molecular epidemiological investigations. Utilising this gene target, we provide additional evidence to support cross-host transmission between different animal hosts.

**Conclusions:**

Our results suggest a history of parasite invasion through colonisation of Australia from hosts across the globe and the potential for cross-host transmission being a common feature of the epidemiology of this neglected pathogen. If this is the case, comparable patterns may exist elsewhere in the ‘New World’. This work provides a basis for expanded molecular studies into mange epidemiology in humans and animals in Australia and other geographic regions.

**Electronic supplementary material:**

The online version of this article (10.1186/s12862-017-1086-9) contains supplementary material, which is available to authorized users.

## Background

Estimated as infecting 110 million humans and more than 100 mammalian species worldwide, *Sarcoptes scabiei* is widespread, burdensome, and has among the widest host range of known parasites [1, 2]. *S. scabiei* infests the epidermis of its hosts, causing a wide range of host immune responses and associated mange disease symptoms (otherwise classified as scabies in humans) including skin irritation, inflammation, hyperkeratosis, alopecia, pruritis, rheumatic heart disease, and secondary bacterial infections [1]. The life cycle of this mite has five developmental stages (egg, larva, protonymph, tritonymph and adult) occurring within the stratum corneum, with durations of life cycle occurring between 7 and 21 days [3–5]. It is most commonly reported in humans, domestic dogs and livestock, with impacts to health, animal welfare and primary production [4, 6, 7]. Transmission of mites between individuals and hosts occurs by direct contact, sharing of contaminated materials or habitat with mites known to survive off its host for up to three weeks [3, 5]. With a wide and expanding wildlife host range, this pathogen is also labelled an emerging infectious disease in North America and Australia [7].

Sarcoptic mange or scabies is a major parasitic disease of indigenous people and their domestic dogs, particularly in northern Australia [8–10]. In remote Aboriginal communities, up to 50% of children suffer from *S. scabiei* infestations, resulting in endemic transmission and severe cases of scabies induced pyoderma [9]. These communities also share communal space with mange infested dogs, a risk for continuous zoonotic transmission [10]. *S. scabiei* has also been documented to infest iconic Australian native species, including wombats (*Vombatus ursinus*, *Lasiorhinus latifrons*) [11], wallabies *(Wallabie bicolor, Macropus agilis)* [12, 13], koala (*Phascolarctos cinereus*) [14], southern brown bandicoot (*Isoodon obesulus*) [15], and dingo (*Canis lupus dingo*) [6]. Among these, wombats appear to experience the most severe pathology with mange associated with population declines exceeding 90% in some instances [16, 17]. Recent studies suggests a rise in mange cases in koalas also, particularly in populations from southern Australia [18].

Despite large numbers of mange cases in these wildlife species, the origins and phylogenetic relationships of *S. scabiei* among host species remains significantly understudied. Only two studies have contributed to the genetic knowledge of Australian-derived mites and these have revealed contrasting results. The first of these studies utilized sequencing of partial *S. scabiei* 12S rRNA gene sequence fragments [19], revealing close sequence similarity between mites from wombats, dogs and humans in Australia [19]. A subsequent investigation examining mites from Australian humans and animals and comparing them to mites from elsewhere, however, highlighted a strong relationship between *S. scabiei* mitochondrial DNA haplotypes based on host and geographic differences [10]. While no new sequences were obtained from Australian animals, most recently, a French study of 12S rRNA and *cox1* gene sequences from *S. scabiei* isolated from humans and dogs supported Skerratt et al.’s [19] original suggestion of a relationship between human, domestic animal and wombat sequences. It is, thus, proposed that occurrence of sarcoptic mange in wombats is the result of past spillover from humans and dogs, potentially from Europe. The exact relationship between mites infesting native Australian wildlife and the endemic strains shared between indigenous human populations and domesticated dogs is currently unknown due to the almost complete absence of any additional sequence data from these sources of *S. scabiei* [20].

Apart from the chronic shortage of genetic information for *S. scabiei*, considerable controversy exists over the choice of gene loci to be used for molecular typing of this pathogenic mite. The most common targets for *S. scabiei* typing studies to date have been the mitochondrial 12S rRNA, 16S rRNA and Cytochrome oxidase 1 (*cox1*) genes. The 12S rRNA gene is highly conserved and does not discriminate well between host species infected by *S. scabiei*, although some studies have used it for this purpose [19, 21]. The 16S rRNA gene is less conserved, though is also limited in its ability to identify genetic distinctions among *S. scabiei* mites separate hosts or locations [22, 23]. *cox1* is the most variable of the three genes, with greater power to distinguish between mites from different host species [20, 24–26]. Nuclear markers have also been investigated including the use of microsatellites [27–30] and phylogenetic analysis of ITS-2 gene sequences [23, 25, 31–34]. The latter studies concluded that *S. scabiei* was a single heterogeneous species with a low level of genetic diversity [23, 25, 31–34]. Microsatellites studies have provided some genetic evidence for cross-species infestation [29], with some limited ability to distinguish mites from different host groups but not by geographic location of isolation [27–30].

In an effort to gain insight into the origins of *S. scabiei* in Australian wildlife and to provide base-line data for studies to investigate the relationship to *S. scabiei* infestations of humans and domesticated animals, we sequenced the mitochondrial genomes of *S. scabiei* mites collected from bare-nosed wombats from Tasmania, New South Wales (NSW) and Victoria (VIC), and koalas from VIC. We used this new genetic information to assess the potential role of *S. scabiei* cross-host transmission to and from marsupial host species following detailed phylogenetic analysis. Because the choice of genetic loci for molecular epidemiology studies has influenced previous studies of *S. scabiei* origins and host specificity, we additionally examined individual mitochondrial genes to aid in the selection of gene targets for downstream investigations into the phylogenetic relationships of mites from a range of hosts and geographic locations, not only for Australian studies but for global comparisons.

## Methods

### Mite collection and DNA extraction

Mites were obtained via skin scrapings during necropsy from Victorian koalas (*N* = 5) and wombats from Victoria (*N* = 2) and Tasmania (*N* = 3) or as a part of routine veterinary care from anaesthetised New South Wales wombats (N = 2). From each individual host, 1–3 mites were pooled for DNA extraction (to obtain sufficient mtDNA for sequencing) following protocol using a DNeasy Blood and Tissue kit (Qiagen). The term “mite” will be used here forth for pooled sequences of several individuals.

### Mitochondrial DNA long range PCR

To amplify full-length mitochondrial DNA sequences, primers were designed to cover four overlapping fragments (Additional file 1). Each long-range PCR was conducted on a thermocycler with a final PCR volume of 20 μL; 5 μL 5X PrimeSTAR® GXL DNA buffer (Takara), 2 μL dNTP mixture (2.5 mM), 1 μL PrimeSTAR® GXL DNA Polymerase (Takara), 1 μL of forward and reverse primers (0.3 μM), 9 μL water and 1 μL of DNA template. Cycling conditions consisted of 35 repeats of 98 °C for 10 s, 50 °C for 20 s and 68 °C for 10 mins. The presence of amplicons of the expected size was visually verified on a 1% TBE agarose gel, prior to purification using QIAquick PCR purification kit (Qiagen). DNA sequences of purified amplicons were determined by paired-end sequencing on a HiSeq4000 (BGI, Hong Kong) at 100 Mb coverage after passing quality control.

### *S. scabiei* mitochondrial genome assembly and annotation

Read mapping was performed in Geneious 9.1.3 [35] using the Geneious mapper method, originally using the four primer pair sequences used for PCR amplification as the reference sequence at 5 iterations, 100% identity and a minimum overlap of the same length of the primers (*cox1* at 22 bp, 12S rRNA at 30 bp, *cob* at 26 bp and *nd4* at 32 bp). Reads were then mapped to the four output contigs at 100 bp overlap, 99% identity and 100 iterations. Contigs produced from this assembly were aligned using MUSCLE alignment [36] in Geneious version 9.1.3 [35]. Assembly of the new mitochondrial genome sequences obtained in this study was confirmed by sequence alignment against the previously published human *S. scabiei* mitochondrial genome (accession number LN874268).

Mitochondrial protein coding genes were annotated by identifying open reading frames (ORF) in Geneious and blastp to confirm ORF identification and protein length. tRNA genes were identified using ARWEN [37]. The two rRNA genes were identified by blastn search of GenBank [38]. DnaSP [39] was used to assess the magnitude selective pressure by dN/dS rations for all protein coding genes.

### Phylogenetic tree analysis

The relationship between the new *S. scabiei* mitochondrial genomes and the available human derived mite was conducted by neighbour-net analysis at 1000 bootstrap replicates using SplitsTree (version 4.14.4) [40], showing only bootstrap values greater than 80. Individual full length gene tree outputs of 16S rRNA, cox1, cox3, cytb and ND4 were constructed with Mr. Bayes [41] in Geneious version 9.1.3, and were then compared to the Mr. Bayes tree output produced by the near full length (NFL) mitochondrial genome. Node tips and clade outputs were then visually compared between the two trees of interest, with associated tip lines drawn between the trees for clarity.

Global *cox1* phylogenetic analysis was completed using the current 12 mitochondrial genomes and the 78 available *cox1* sequences in GenBank. All *cox1* sequences were unified by trimming to 387 bp to allow alignment of sequences of the same length. Phylogenetic tree construction on the resulting 90 sequences was performed by Mr. Bayes and rooted with *Otodectes cynotis* mitochondrial genome (KP676688). Bootstrap values greater than 80 were shown.

## Results

### *S. scabiei* mitochondrial genome assembly and annotation identify new haplotypes in Australian marsupials

Successful full-length mitochondrial genome assembly was confirmed for all samples (accession numbers MF083732-MF083743). Neither single nor pooled DNA extraction played a significant role in amplification or exhibited sequencing errors. The mitochondrial genome size ranged between 13,830 bp and 13,926 bp with variations in length largely owing to the non-coding repeat region of “ATs”. The mitochondrial genome GC content was comparable amongst all samples, and all new *S. scabiei* from koala and wombats were >98% similar to the mite genome from humans (Table 1).

**Table 1:** Details of new mitochondrial genomes, size, GC content and their sequence similarity to the human *S. scabiei* mite (LN874268)

Sample Name	Host (Location)	Pooled/Single Mite	Genome Size (bp)	GC %	% Similarity to LN874268
DW02	Wombat (Narawntapu, Tasmania)	Single	13,911	19.21	99.44
S1	Wombat (South Swansea, Tasmania)	Single	13,839	19.37	99.03
B1	Wombat (Brighton, Tasmania)	Single	13,887	19.23	99.47
N11	Wombat (Central Coast, New South Wales)	Pooled	13,845	19.39	98.62
N22	Wombat (Murrys Run, New South Wales)	Single	13,900	19.32	98.98
W2	Wombat (Kangaroo Ground, Victoria)	Pooled	13,856	19.33	99.06
VW	Wombat (Arthurs Creek, Victoria)	Pooled	13,848	19.35	99.16
K2	Koala (Koonoomoo, Victoria)	Pooled	13,926	19.29	99.43
K4	Koala (Koonoomoo, Victoria)	Pooled	13,828	19.39	99.18
K5	Koala (Sandy Point, Victoria)	Pooled	13,830	19.40	99.21
K7	Koala (Koonoomoo, Victoria)	Pooled	13,854	19.35	98.86
K8	Koala (Koonoomoo, Victoria)	Pooled	13,846	19.37	99.39

Thirteen protein coding genes were identified in all 12 mitochondrial genomes using the invertebrate mitochondrial genetic code. tRNA annotation (ARWEN) resolved the presence of 16 of the 22 tRNA genes, with a further four added (*trnQ*, *trnR*, *trnP* and *trnF*) based on a visual inspection of the mitochondrial genome sequences and sequence identity to the previously annotated human *S. scabiei* mitochondrial genome (LN874268) [42]. Two invertebrate tRNA genes (*trnA* and *trnY*) were not identified, however these were also missing in the human *S. scabiei* mitochondrial genome (LN874268) [42].

Sequence analysis of the wombat and koala mite mitochondrial genomes, after removing the repeat region variation between *trnS1* and *trnF*, revealed the presence of 11 unique haplotypes that shared between 99.1% and 100% sequence identity against the human *S. scabiei* mitochondrial genome. All NFL mitochondrial genomes were the same length when aligned (13,822 bp). Between the new genomes, only two koala mites (K4 and K7) shared 100% sequence similarity. The highest number of differences occurred between two wombat mites, N11 and DW02, from NSW and Tasmania respectively (171 nucleotides). The NFL mitochondrial genome of the human *S. scabiei* mite had the highest number of differences to a NSW wombat mite (N11) (178 nucleotide) and the lowest number of differences to a Victorian koala (K8) (83 nucleotides).

### Phylogenetic analysis reveals a range of *S. scabiei* haplotypes among koalas and wombats

**Fig. 1:**
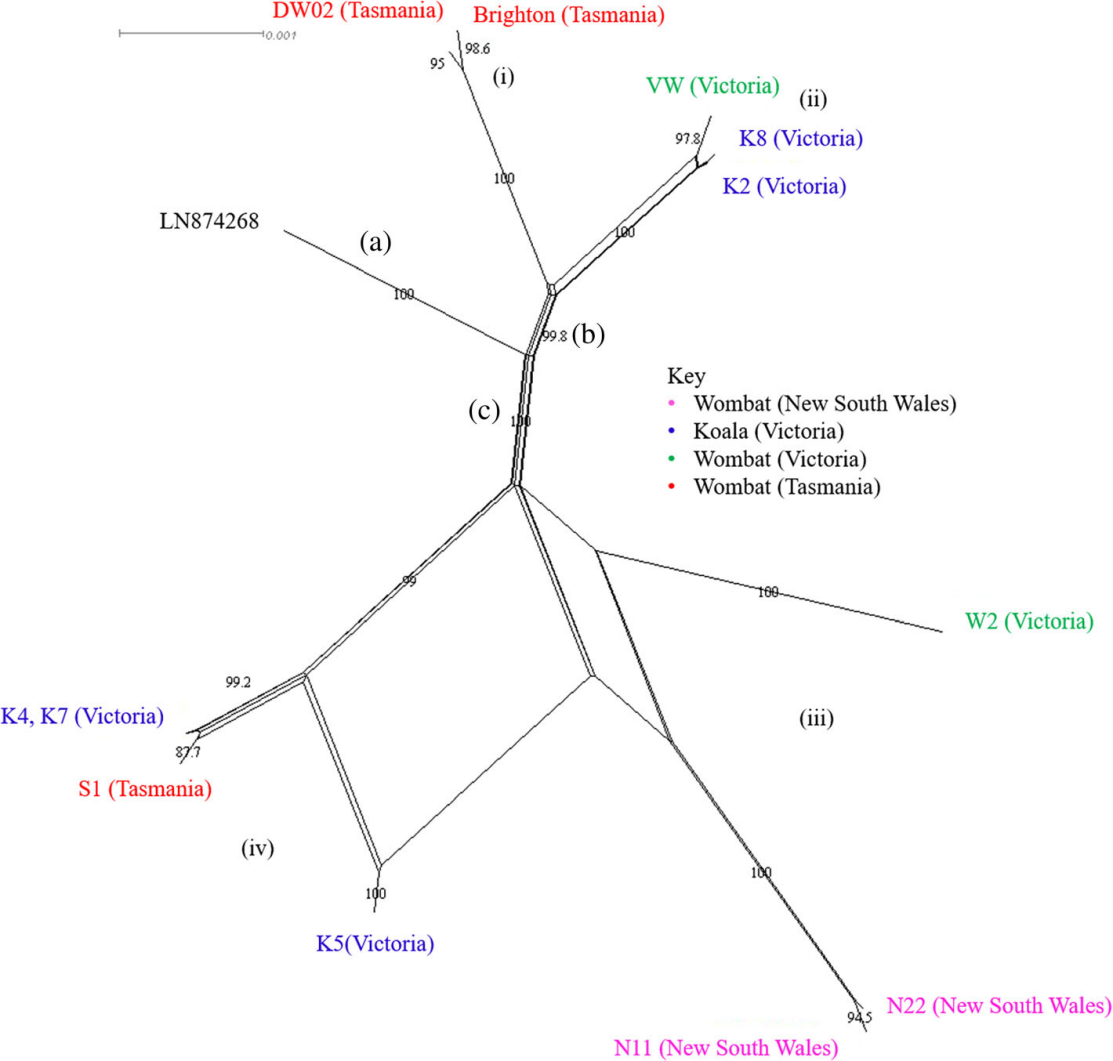
SplitsTree anlaysis of new near full-length mitochondrial genomes for koala and wombat *S. scabiei*. Three major clades are identifed by splits: **a** the human mite LN874269, **b** marsupial mites from Tasmania and Victoira and, **c** marsupial mites from New South Wales, Victoria and Tasmania. Four subclades are further recognized, labeled (i-iv)

SplitsTree phylogenetic analysis of complete *S. scabiei* near full-length mitochondrial genomes revealed three major clades (bootstrap support >0.8), (Fig. 1); one is exclusively comprised of the human mite (LN874268) and the remaining two are a mix of marsupial mites. Each marsupial lineage could be further subdivided into two smaller clades comprising: (i) wombat mites from Tasmania (DW02 and Brighton), (ii) wombat (WV) and koala mites (K8 and K2) from Victoria, (iii) wombat mites from New South Wales (N11 and N22) and Victoria (W2), and; (iv) mites from a single wombat from Tasmania (S1) and koalas from Victoria (K4, K5 and K7). With the exception of subclade iii, all subclades are strongly supported by the length of parallel splits and their corresponding bootstraps. No clear host or geographical pattern was evident between the two major marsupial mite clades. We conducted a range of phylogenetic analyses all emitting the same result (Additional file 2).

### Co-phylogenetic comparisons show individual mitochondrial genes vary in their utility for molecular epidemiological investigations

The expanded set of mitochondrial genome sequences for *S. scabiei* generated in this study enabled an evaluation of several mitochondrial genetic markers for molecular epidemiology studies. DnaSP [43] analysis revealed that (i) every mitochondrial gene is under negative selection with *atp8* to be the closest to neutral selection, (ii) *nd4* had the highest number of parsimony informative sites and, (ii) *cytb* identified the largest number of different haplotypes (Table 2).

**Table 2:** Comparison of *S. scabiei* mitochondrial genes from koala and wombat hosts showing the number of informative sites, haplotypes and the magnitude of natural selection (dN/dS)

Gene locus	Length (bp)	Number of parsimony informative sites	dN/dS	Number of haplotypes
12S rRNA	657	4	NA	7
16S rRNA	1040	13	NA	8
*atp8*	159	4	0.705	4
*cox1*	1551	17	0.040	9
*cox2*	751	9	0.031	6
*cox3*	783	13	0.022	8
*Cytb*	1101	16	0.066	10
*nd1*	900	11	0.055	6
*nd2*	928	12	0.245	6
*nd3*	354	3	0.105	7
*nd4l*	255	3	0.000	3
*nd4*	1297	21	0.132	8
*nd5*	1626	20	0.040	7
*nd6*	441	4	0.000	5

**Fig. 2:**
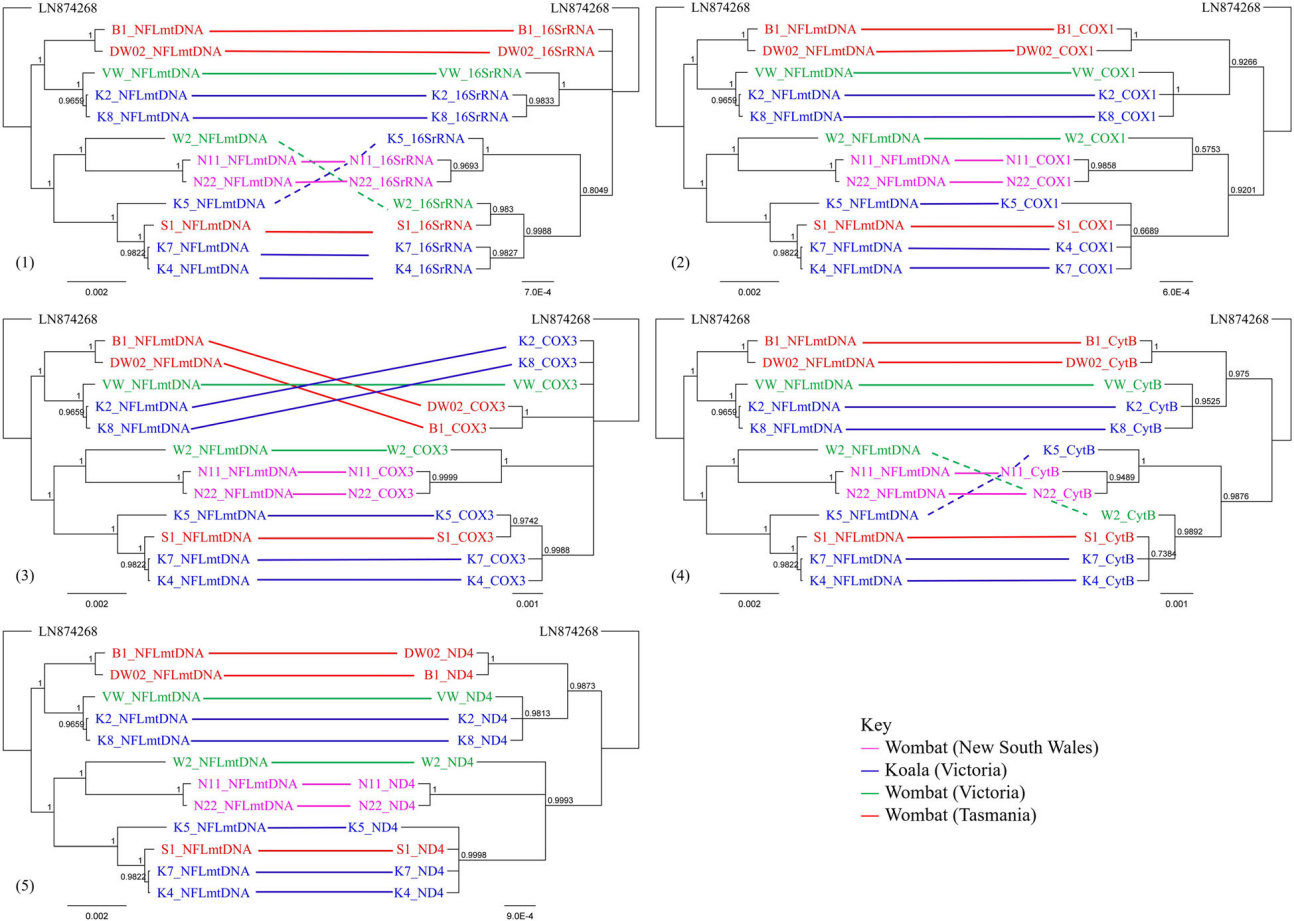
Comparison of tree output of NFL mitochondrial genomes to full-length individual gene trees. Comparison to NFL genomes include: **1** 16S rRNA gene, **2** *cox1*, **3** *cox3*, **4** *cytB* and, **5** *nd4*. All trees were similar but only *cox1* and *nd4* produced identical clustering

Using the five genes with the greatest number of unique haplotypes (16S rRNA 8, *cox1* 9, *cox3* 8, *cytb* 10 and *nd4* 8), a co-phylogenetic comparison of their trees was made to the NFL phylogeny (Fig. 2). All trees were rooted with the human mite (LN874269) to allow comparisons of clade construction and analysis of how the new haplotypes relate to each other. The phylogenetic trees constructed from each of the five genes portrayed the three major clades shown by analysis of the NFL mitochondrial genomes. Noticeably, the phylogenetic trees constructed on two of these genes (*cox1* and *nd4*) also revealed the same subclade structure. For *cox3* and 16S rRNA genes, the low number of haplotypes identified revealed less resolution of putative ancestral linages. *Cytb* and 16 s rRNA genes differed with the position of K5 and W2 alternating between with the two NSW wombat mite clades and the VIC koala and Tasmanian wombat mite clade. The change in relative position of K5 and W2 in the latter trees expands a subclade that is predicted to include only wombat sequences based on NFL mitochondrial sequence analysis to also include sequences from koalas.

### cox1 analysis reveals that Australian marsupials share *S. scabiei* haplotypes with a diverse range of globally distributed mites

**Fig. 3:**
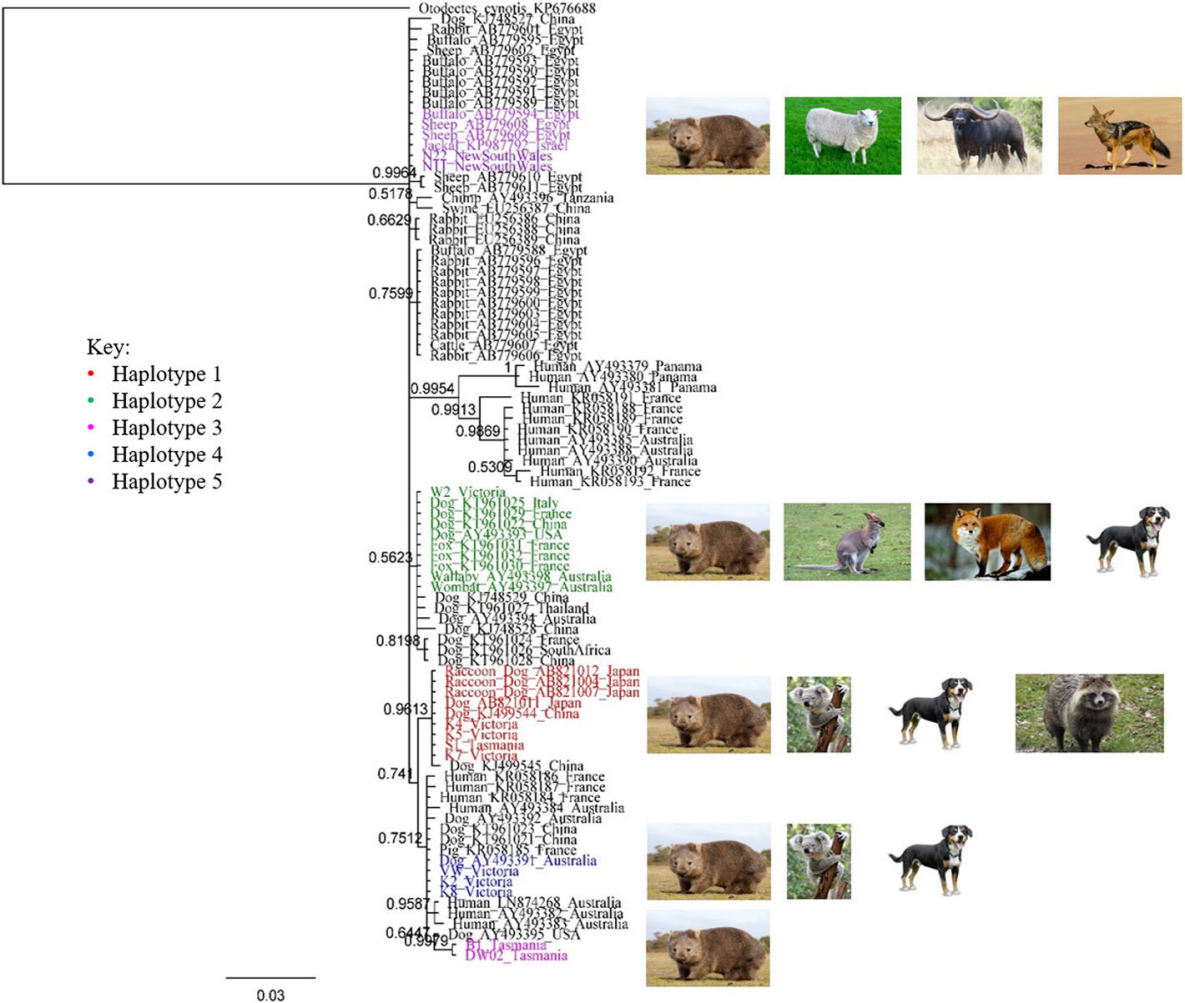
Phylogenetic analysis of global *cox1* sequences. Comparison of 78 available *cox1* sequences in GenBank were used to construct a global phylogenetic tree, including the current Australian mitochondrial genomes, with *Otodectes cynotis* mitochondrial genome (KP676688) as the outgroup. Sequence comparison and clade outgroups identified five haplotypes which the new marsupial mite sequences were identical to as shown by colour and images of hosts for each haplotype. Each sequence from GenBank is labels as Host_Accession Number_Location

We combined our new *cox1* sequences with other global *cox1* haplotypes from across the globe [20, 24, 44] to perform a phylogenetic analysis to elucidate the relationships between Australian mites and those from animals and humans elsewhere. Sequence comparison and clade outgroups identified five haplotypes: (i) K7, S1, K4 and K5 were identical to a dog mite and three racoon dog mites from Japan (AB821011, AB821004, A821007, AB821012), (ii) W2 was identical to a dog mite from China (KT961022), a dog mite from Italy (KT961025), a single dog mite and three fox mites from France (KT961029, KT961030, KT961031, KT961032), a dog mite from the USA (AY493393) and a wombat mite and wallaby mite from Australia (AY493397 and AY493398), (iii) B1 and DW02 were the same and unique to everything else, (iv) VW, K2 and K8 are identical to a single dog mite from Australia (AY493391) and, (v) N11 and N22 were identical to a jackal mite, a buffalo mite and two sheep mites from Egypt (KP987792, AB779594, AB779608, AB779609). From the phylogenetic tree (Fig. 3) haplotypes containing the Tasmanian wombats (B1 and DW02) and Vic marsupials (VW, K2 and K8) are the only sequences that sit within other human sequences from Australia and France and dog sequences from China. All other human sequences are within its own distinct major clade.

## Discussion

Pathogen dispersal and spillover likely underscore global infestations of *S. scabiei* among many animal species, including Australian wildlife [45]. However, a dearth of genetic information for this mite has hindered molecular epidemiological investigations. We tackled this problem with mitochondrial genome sequencing of *S. scabiei* across a broad geographic range of two impacted marsupial host species. The comparison of NFL genome assemblies showed unique haplotypes infesting koalas and wombats across south-eastern Australia. Despite high NFL sequence similarity (>99%), our analysis revealed 11 unique mitochondrial haplotypes, with only two koala mites sharing 100% sequence identity.

The presence of at least two major evolutionary lineages of *S. scabiei* occurring among wombats in Tasmania, and wombats and koala mites from Victoria suggests a polyphyletic origin may exist for *S. scabiei* in Australia. The absence of major clade separation between wombats and koala mites suggests a level of pathogen generality, indicative of multiple cross-species transmission events. Although we did not sample other animals in this study, we think it is reasonable to hypothesise that these cross-species transmission events may also include other animals such as domestic and wild dogs and invasive foxes.

Although more sampling is clearly required to address this question conclusively, our observations supports the hypothesis that *S. scabiei* was first introduced to Australian wildlife in the time following European colonisation. This is based on (i) significant sequence similarity in the NFL mitochondrial genomes of the marsupial mites sampled in this study and; (ii) the single gene molecular typing results that reveal that *cox1* haplotypes are shared between marsupial mites and those from a diverse range of animal hosts in the rest of the world, Indeed this is not a new suggestion [11, 19–21], but our data provides the strongest support yet for this hypothesis. Historical records of mange disease in humans and domestic dogs from Tasmania date back to as early as the 1820s [46, 47], suggesting that Australian wildlife have been infested by *S. scabiei* for at least 200 years. The main question appears to simply be how long before then were mites introduced. Pre-European hypotheses must still be considered (albeit weakly) plausible, including introduction through aboriginal colonisation (ca. 50,000–60,000 years ago), introduction of the dingo (ca 4000 years ago), or deeper evolutionary origins. If *S. scabiei* mitochondrial genome evolutionary rates are similar to that of other invertebrates (i.e. 10^−6^ substitutions per year [48, 49]), then the evolutionary timescale of Australian marsupial mite divergence would be likely greater than >200 years. This prediction, however, does not take into account potential changes to this rate associated with spillover into a new host and the obvious limitations of making these interpretations on such a small sample set. Although a less parsimonious explanation, we also cannot obviously exclude the potential for mitochondrial capture and/or selective sweep phenomena to potentially mask the genetic signature of mites that may have been present in Australian animals prior to European colonisation. The resolution of the origins of *S. scabiei* in Australia will require sampling of mites from a greater time span from humans, marsupials and other hosts or a significant expansion in genomic sequence data for *S. scabiei*.

The availability of an extended set of mitochondrial sequences beyond the first initial human *S. scabiei* mitochondrial genome sequence provided an opportunity to re-assess the suitability of different mitochondrial genetic markers for detailed molecular epidemiology. Our research confirms that the two mitochondrial rRNA genes (12S and 16S) which have been used to infer origins of *S. scabiei* to Australia in the past, provide insufficient resolution for such assessments. The 16S rRNA gene, which is the more variable of the two genes (eight haplotypes identified in this study), can differentiate mites from differing host families, but cannot separate host species and location specific *S. scabiei* [20, 22], or preserve the resolution of ancestral origin when compared to the NFL mitochondrial genome. It has been suggested that *cox1* should be used as mitochondrial genetic marker due to the high variability and its ability to distinguish between biogeographical regions, host families, genera, species and even subspecies [20, 22, 50, 51]. Here, we also support this suggestion, with the phylogenetic reconstruction of the gene preserving an identical tree to the NFL mitochondrial genome. In the absence of available NFL genetic data for downstream molecular epidemiological investigations of *S. scabiei* in humans and animals, our analysis would appear to support the continued use of *cox1* as a gene target for molecular typing.

To explore this further, we combined the new *cox1* sequences from this study with new haplotypes from Europe [24] and other global haplotypes [20] to interrogate phylogenetic associations among hosts globally. Confirming our previous research [20], we found the Australian *S. scabiei* haplotypes share sequence identity with mites from non-human hosts in Europe, the Middle-East and Asia. These multiple geographic associations among a variety of host species potentially supporting multiple introduction events of *S. scabiei* from differing geographic regions into Australia, as indicated by the two mitochondrial genome clades shown in this study and the presence of multiple genetically distinct mites in Tasmania, some of which are also found on the mainland. Cross-host transmission has previously been demonstrated in the case of wombat to human transmission [52] and other experimental cross-infestations [31, 45, 53]. While clear genetic evidence for cross-host transmission between Australian marsupials and other hosts is lacking at this stage, the observation of shared *cox1* gene sequences (and similar NFL mitochondrial genome sequence data from this study) between Australian marsupial species suggests this is very likely. This observation alone highlights the need for additional precautions in the management of mange between different Australian wildlife and a renewed emphasis on infection control practices for stakeholders involved in their care.

## Conclusions

This study is the first to sequence the mitochondrial genomes of *S. scabiei* in Australian marsupials. In doing so, we revealed a high level of sequence similarity among the marsupial mite sequences supporting the likely transmission of these mites between marsupial hosts affected. A greater repertoire of mites from more marsupial hosts spanning across Australia and other hosts globally would be beneficial. Mites from dingos, foxes and wild dogs in Australia would be particularly ideal as they likely contribute to the intra- and inter-specific transmission dynamics of *S. scabiei* on mainland Australia. Furthermore, an expansion of sample collection over time and geographical areas would also be beneficial for estimating rates of evolution, allowing evolutionary divergence to be better assessed. Our comparison of gene trees confirmed the use of *cox1* as the most informative gene (when sequenced in full length) for phylogenetic comparisons. An expansion of such studies leveraging the genetic data provided in this study is expected to provide further insight into the global dissemination of this widespread and neglected human and animal pathogen.

## Additional files

Additional file 1: Primer details for long range PCR. The four long range PCR fragments spaning across the mitochondrial genome are 4.4 kb, 4.0 kb, 3.8 kb and 1.7 kb long. These primers are positioned on four main genes; 12S rRNA gene, *nd4*, *cob* and *cox1*. (DOCX 13 kb)

Additional file 2: Additional phylogenetic trees constructed for new NFL mitochondrial genomes. Five different phylogenetic trees were constructed; 1) Log-Det NeightbourNet, 2) Raw (Uncorrected) proportional distance and Neighbour-Net, 3) Tree-inference method with Jukes-Cantor (1969) distances, 4) Neighbour-joining tree using uncorrected distances and 5) Neighbour-joining tree with bootstrap values. (DOCX 343 kb)

## Abbreviations

NFL: Near full length
NSW: New South Wales
ORF: Open reading frame
PCR: Polymerase chain reaction
rRNA: ribosomal RNA
*S. scabiei*: *Sarcoptes scabiei*
tRNA: transfer RNA
VIC: Victoria
