# Supplementary material for: Mitochondrial genome sequencing reveals potential origins of the scabies mite Sarcoptes scabiei infesting two iconic Australian marsupials
Source: BMC Evol Biol. 2017 Nov 28;17:233. doi: 10.1186/s12862-017-1086-9 (PMC5706379; doi:10.1186/s12862-017-1086-9)
Supplement: Supplementary file 1 — Primer details for long range PCR. The four long range PCR fragments spaning across the mitochondrial genome are 4.4 kb, 4.0 kb, 3.8 kb and 1.7 kb long. These primers are positioned on four main genes; 12S rRNA gene, nd4, cob and cox1. (DOCX 13 kb) [file 12862_2017_1086_MOESM1_ESM.docx]

**Table S1**

| Fragment Length | Gene direction | Primer 1 (5’ – 3’) | Gene direction | Primer 2 (5’ – 3’) |
| --- | --- | --- | --- | --- |
| 4.4kb | 12S rRNA forward | CAAGTTCCTGTGAATATATAGAAAACCGCC | *cox1* forward | GGACACCCGGAAGTTTACATTC |
| 4.0kb | *nd4* forward | CCAAAAGCTCATGTAGAAGCTCCTCTAGAAGG | *cob* reverse | TAGTTACTCCTACTCATATTCAACC |
| 3.8kb | *nd4* reverse | CCTTCTAGAGGAGCTTCTACATGAGCTTTTGG | 12S rRNA reverse | GGCGGTTTTCTATATATTCACAGGAACTTG |
| 1.7kb | *cob* forward | GGAACTGAACGAAGAATAGCATAAGC | *cox1* reverse | GAATGTAAACTTCCGGGTGTCC |
