# Supplementary material for: Mitochondrial genome sequencing reveals potential origins of the scabies mite Sarcoptes scabiei infesting two iconic Australian marsupials
Source: BMC Evol Biol. 2017 Nov 28;17:233. doi: 10.1186/s12862-017-1086-9 (PMC5706379; doi:10.1186/s12862-017-1086-9)

**Additional file 2:** Additional phylogenetic trees constructed for new NFL mitochondrial genomes.

1. Log-Det NeightbourNet


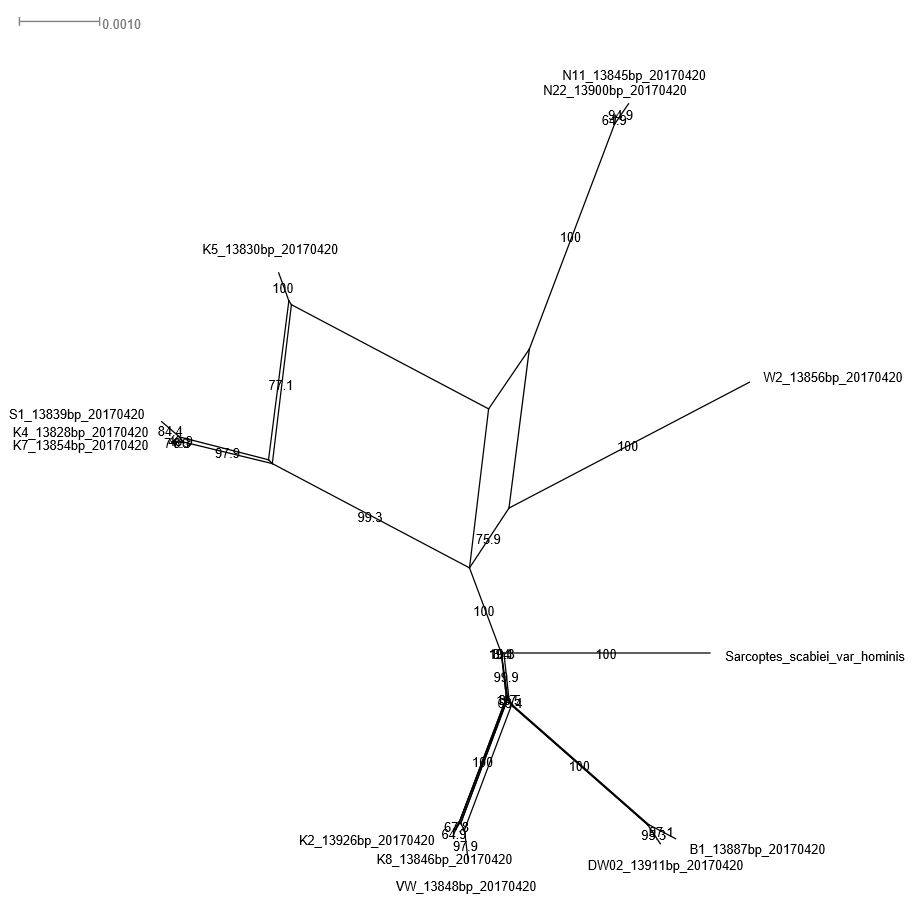


1. Raw (Uncorrected) proportional distance and Neighbour-Net


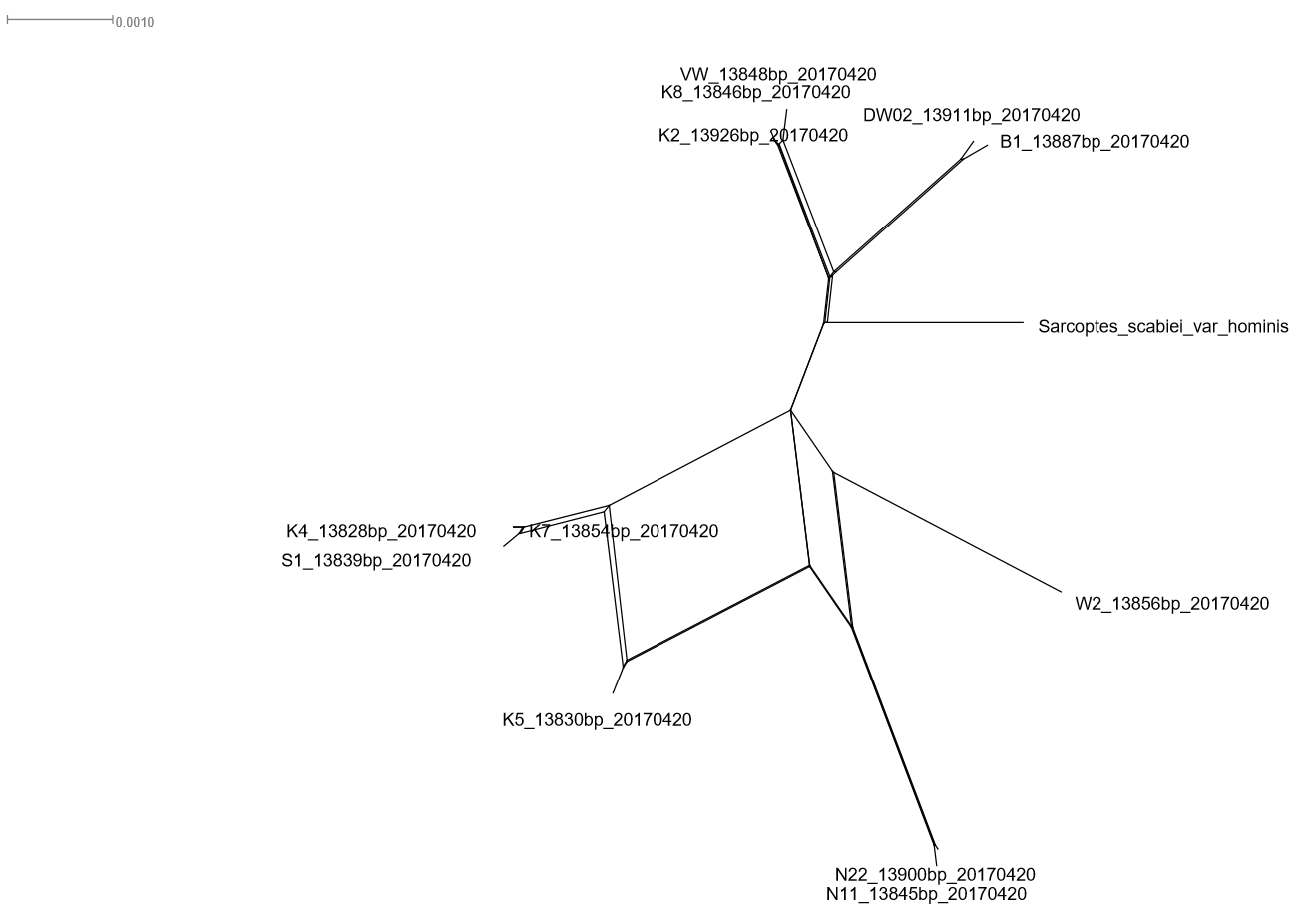


1. Tree-inference method with Jukes-Cantor (1969) distances


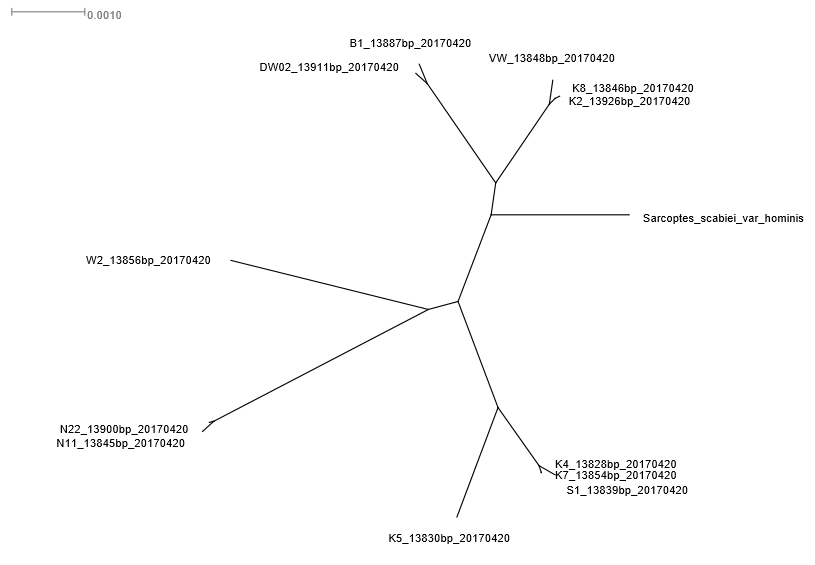


1. Neighbour-joining tree using uncorrected distances


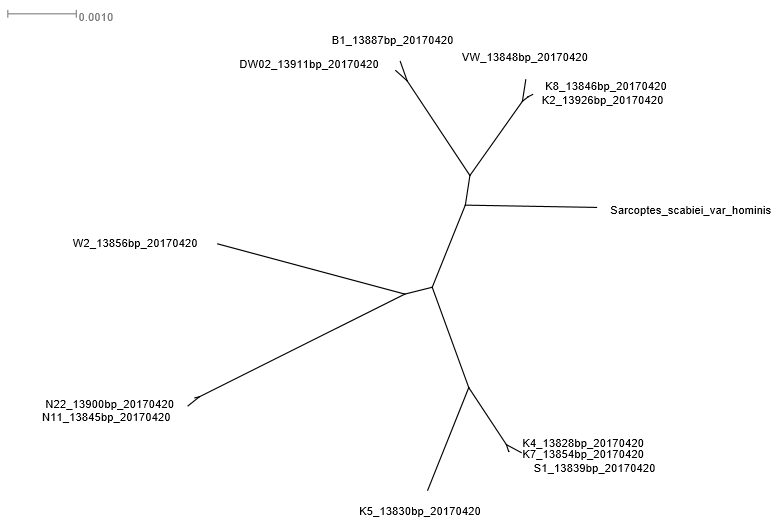


1. Neighbour-joining tree with bootstrap values


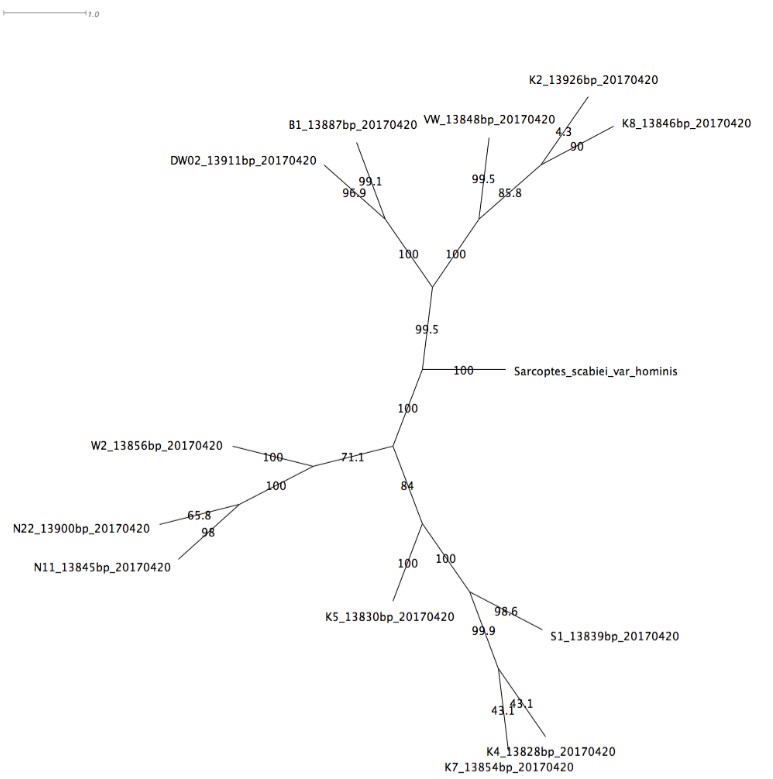

Supplement: Supplementary file 2 — Additional phylogenetic trees constructed for new NFL mitochondrial genomes. Five different phylogenetic trees were constructed; 1) Log-Det NeightbourNet, 2) Raw (Uncorrected) proportional distance and Neighbour-Net, 3) Tree-inference method with Jukes-Cantor (1969) distances, 4) Neighbour-joining tree using uncorrected distances and 5) Neighbour-joining tree with bootstrap values. (DOCX 343 kb) [file 12862_2017_1086_MOESM2_ESM.docx]
